# East London Hospital for Children

**Published:** 1905-04-15

**Authors:** 


### EAST LONDON HOSPITAL FOR CHILDREN.

### OPENING OF THE NEW WHOOPING COUGH WARDS.

Amid signs of festivity, which pervaded the neighbourhood of Glamis Road and the adjoining artisans' dwellings, the Princess Louise Augusta of Schleswig-Holstein opened the new Whooping Cough Wards, at the East London Hospital for Children, on Saturday afternoon (8th inst.). The out-patient department, in which the ceremony took place, was well filled by a large number of friends of the supporters, and a display of bunting, tastefully arranged flowers and palms, with the cheerful strains of the band of the 1st Life Guards, added to the gay effect. The Princess, who was welcomed by the Mayor and Aldermen of Stepney, and by a large Reception Committee, accepted a bouquet of roses and lilies of the valley from Miss Adelaide Row, after which she inspected the new block. This contains 12 beds, in two wards, and accommodation for half that number of nurses. The nurses' dining-room is on the entrance floor, and this, like the bedrooms, is simply and tastefully furnished in fumed oak. The rooms and wards are arranged so as to secure a good current of air, and there is ample light from the windows. Closed central stoves have been used in the wards, and open fireplaces in the corridors. Electric light has been installed. There is an outside iron staircase by which the patients will be brought into the block, and which also serves as a fire-escape and gives access to the roof.

Prayers having been said by the Bishop of Stepney, an address was read by the chairman of the hospital, Colonel Charles Needham, thanking the Princess for her kindness in opening the new wards, which, he explained, formed a new departure in the history of the hospital. Up to the present infectious cases had been either sent to a fever hospital or delegated to the isolation block, and the mortality from neglected cases of whooping cough being abnormally high in the East End of London, it was considered that a great boon would be conferred on the public if serious cases, admitted at once, were treated in properly equipped wards. This consideration had led the Board to construct the two wards, with the corresponding accommodation for the nurses, in connection with the isolation block, at a cost of over £10,000 for the entire building, of which £3,350 had been granted by annual allowances from King Edward's Hospital Fund, and £1,150 had been received from other sources. Of the £5,500 still required, £3,500 had been already promised, conditionally on the balance (£2,000) being subscribed by June 30th, 1905. The Board of Management, Colonel Needham added, were greatly in hopes that the ceremony might give publicity to the existence and needs of the hospital, which from its isolated position was little known to the general public, and which received little local support owing to the extreme poverty of the neighbourhood. The architect, Mr. W. Campbell Jones, A.R.I.B.A., having presented the Princess with the key of the block, her Highness declared the whooping cough wards open, adding that this not being the first time she had visited the hospital, she could say what an enormous benefit and blessing the new wards would be if carried on on the same lines as the rest of the institution. The Bishop of Stepney having pronounced the Benediction, the Princess received purses in aid of the New Buildings Fund, and a vote of thanks, proposed by Sir Harry S. Samuel, M.P., and seconded by the Mayor of Stepney, closed the proceedings. After the ceremony the Princess visited the wards, and named a bed endowed by "The *Gentlewoman* Children's Salon"—an association of children of the rich working for children of the poor—in the Princess May Ward.

